# RNAi-mediated silencing of the HD-Zip gene *HD20* in *Nicotiana attenuata* affects benzyl acetone emission from corollas via ABA levels and the expression of metabolic genes

**DOI:** 10.1186/1471-2229-12-60

**Published:** 2012-05-01

**Authors:** Brenda Raud, Raquel L Chan, Ian T Baldwin, Gustavo Bonaventure

**Affiliations:** 1 Department of Molecular Ecology, Max Planck Institute for Chemical Ecology, 07745, Jena, Germany; 2 Instituto de Agrobiotecnología del Litoral, Universidad Nacional del Litoral, CONICET, CC 242 Ciudad Universitaria, 3000, Santa Fe, Argentina

## Abstract

**Background:**

The *N. attenuata HD20* gene belongs to the homeodomain-leucine zipper (HD-Zip) type I family of transcription factors and it has been previously associated with the regulation of ABA accumulation in leaves and the emission of benzyl acetone (BA; 4-phenyl-2-butanone) from night flowers. In this study, *N. attenuata* plants stably reduced in the expression of *HD20* (ir-*hd20*) were generated to investigate the mechanisms controlling the emission of BA from night flowers.

**Results:**

The expression of *HD20* in corollas of ir-*hd20* plants was reduced by 85 to 90% compared to wild-type plants (WT) without affecting flower morphology and development. Total BA emitted from flowers of ir-*hd20* plants was reduced on average by 60%. This reduction occurred mainly at the late phase of BA emission and it was correlated with 2-fold higher levels of ABA in the corollas of ir-*hd20* plants. When a 2-fold decline in ABA corolla levels of these plants was induced by salt stress, BA emissions recovered to WT levels. Supplying ABA to WT flowers either through the cuticle or by pedicle feeding reduced the total BA emissions by 25 to 50%; this reduction occurred primarily at the late phase of emission (similar to the reduction observed in corollas of ir-*hd20* plants). Gene expression profiling of corollas collected at 12 pm (six hours before the start of BA emission) revealed that 274 genes changed expression levels significantly in ir-*hd20* plants compared to WT. Among these genes, more than 35% were associated with metabolism and the most prominent group was associated with the metabolism of aromatic compounds and phenylpropanoid derivatives.

**Conclusions:**

The results indicated that regulation of ABA levels in corollas is associated with the late phase of BA emission in *N. attenuata* plants and that HD20 affects this latter process by mediating changes in both ABA levels and metabolic gene expression.

## Background

Many plant species emit floral scents as long-distance attraction cues for pollinators, in particular moths, that search and visit flowers at night. The attraction of moths and other animal pollinators have important implications for plant reproductive success; floral scents are important in determining seed or fruit set in non-selfing plants and the frequency of outcross in selfing plants [1-4]. Thus, the biosynthesis and release of floral scents have important ecological and agricultural implications and the molecular mechanisms underlying the regulation of floral scent production are now beginning to be unraveled [5,6].

Floral scents are usually complex mixtures of small volatile molecules and the most prevalent compounds in these mixtures are monoterpenoids, sesquiterpenoids, phenylpropanoids, benzenoid compounds and fatty acid derivatives [1]. Volatile compounds can be either emitted from several parts of the flower or from a specific floral part [7]. The expression of genes encoding floral scent biosynthetic enzymes is temporally and spatially regulated during flower development [1,8]. In most cases studied so far, the expression of these genes correlates with the emission of the corresponding volatile compound indicating that these volatiles are *de novo* synthesized. Thus, scent production and emission are commonly regulated at the transcriptional level. Similar observations have been made in regard to the induction of genes involved in floral pigment production [9].

*Nicotiana attenuata*, a night-flowering tobacco that germinates after fires in the Southwestern United States, normally produces flowers that open at night and release benzyl acetone (BA; 4-phenyl-2-butanone) to attract night-active hawk moth pollinators (*Manduca quinquemaculata* and *M. sexta*)[10]. *N. attenuata* is a fully self-compatible species however it produces more than 30% of its seed from opportunistic out-crossing performed by pollinators [2,11]. BA is the main constituent of the *N. attenuata* floral bouquet [10] and it begins to be emitted from the corolla limb in the evening [10,12]. The emission of BA is synchronized with the development of the flower and it starts as the corolla limb opens [12]. At present, the mechanisms that control the biosynthesis and release of BA from corollas are largely unknown. The BA biosynthesis pathway remains elusive, however, the chemical backbone may derived from the shikimate pathway [1] as *N. attenuata* plants with reduced expression of *CHALCONE SYNTHASE 1* (*CHAL1*) are deficient in BA emission [2].

The homeodomain-leucine zipper (HD-Zip) family of transcription factors (TFs) is a plant-specific family of TFs in which the HD and Zip domains are combined in a single polypeptide [13]. The HD is responsible for the specific binding to DNA and the Zip domain acts as a dimerization motif; dimerization is a prerequisite for binding to the phylogenetically conserved target sequence CAAT (A/T) ATTG [14,15]. The HD-Zip family can be divided into four subfamilies (I to IV) according to the sequence similarity of the HD and Zip domains and to additional structural features outside these domains. The function of HD-Zips type-I has been associated to the regulation of development in response to changes in the environment [13,16-26].

The *N. attenuata HD20* gene was originally identified as an HD-Zip type-I whose expression is induced by multiple stress-associated stimuli including drought and wounding [27]. In a previous study, we reduced the expression of *HD20* by virus induced gene silencing (VIGS) and we demonstrated that *HD20* plays not only a positive role in ABA accumulation in leaves during water stress but also in the emission of benzyl acetone (BA) from night flowers [27]. However, the *HD20*-dependent mechanisms underlying the reduction in BA emission were not studied. Here we investigated these mechanisms by generating transgenic *N. attenuata* plants stably reduced in the expression of *HD20.* These plants were characterized by using a combination of volatile, phytohormone and gene expression profiling approaches to investigate in more detail the process of BA emission.

## Results

### *HD20* mRNA expression in corolla during development

**Figure 1:**
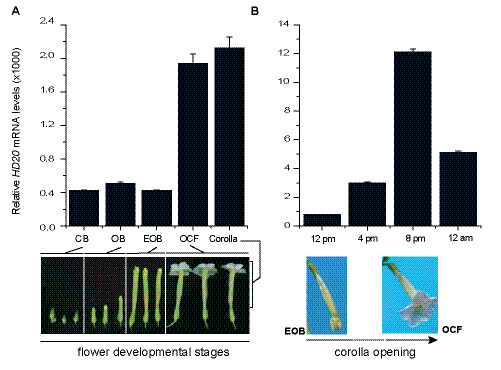
**Analysis of*HD20*transcript levels during flower development.** Total RNA was extracted from both, flowers at different developmental stages and corollas of WT *N. attenuata* plants. Total RNA was reversed transcribed and the *HD20* mRNA levels were quantified by qPCR. Levels are expressed as relative units to the levels of the reference *EF1A* mRNA as calculated by the ΔCT method (*n* = 3, bars denote ± SE). **(A)***HD20* transcript levels were quantified in closed buds (CB), opened buds (OB), elongating opened buds (EOB), opened corolla flowers (OCF), and isolated corollas. Samples were harvested at 5 pm. **(B)***HD20* transcript levels were quantified in isolated corollas at different times during corolla opening.

To investigate in detail the regulation of *HD20* mRNA expression during flower and corolla development in WT plants, the levels of this transcript were first quantified at progressive stages of flower development. *HD20* mRNA was detected in all flower stages and the levels increased as the flower entered the opened corolla stage (opened corolla flower (OCF); Figure 1a). The expression of *HD20* was then quantified during the opening of the corolla, which is a continuous process that starts in the afternoon (between 12 pm and 4 pm) and completes at 8 to 9 pm (see below). *HD20* mRNA levels were quantified at 12 pm, 4 pm, 8 pm and 12 am. The transcript levels increased steadily during the afternoon to peak at 8 pm and to decline at 12 am (Figure 1b).

### Transformation of N. attenuata plants to reduce HD20 expression

**Figure 2:**
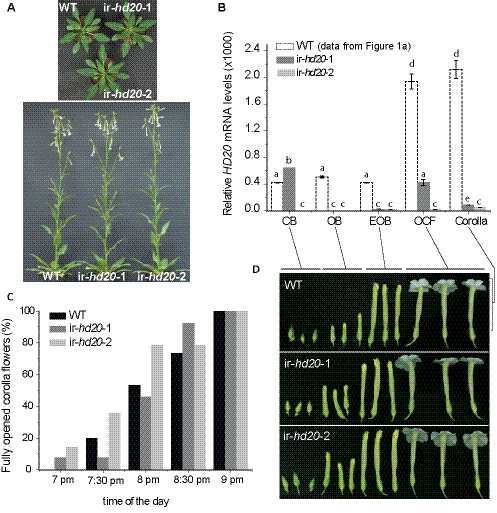
**Morphology and development of ir-*hd20 N. attenuata*plants. (A)** Morphology of ir-*hd20* and WT plants in early rosette and elongated stages. **(B)** Analysis of *HD20* mRNA expression in flowers of ir-*hd20* and WT plants at different developmental stages. The data for WT plants is identical as for Figure 1a and was included only for comparison. Samples were harvested at 5 pm. *HD20* transcript levels were quantified as detailed in legend of Figure 1. Different letters denote significant differences; univariate ANOVA, *F*_*14,44*_ = 26.64 *P* < 0.001 followed by a LSD *post-hoc* test *P* < 0.01 (different letters denote significant differences). **(C)** Kinetic of flower opening in ir-*hd20* and WT plants (*n* = 10). **(D)** Morphology of flowers from ir-*hd20* and WT plants at different developmental stages (CB: closed bud; OB: opened bud; EOB: elongating opened bud; OCF: opened corolla flower).

To investigate the mechanisms mediated by *HD20* and controlling the emission of BA from flowers, stably transformed *N. attenuata* plants with reduced levels of *HD20* expression were generated by inverted-repeat (IR) gene silencing. These lines were named ir-*hd20* and two homozygous independently transformed lines (ir-*hd20*-1 and ir-*hd20*-2) carrying a single T-DNA insertion (Additional file 1) were selected and used for all the experiments in this study (Figure 2a; see Materials and Methods for a detailed description about the generation of these plants). The efficiency of gene silencing in the flowers and corolla of these lines was evaluated by the quantification of *HD20* transcript levels at different developmental stages. The levels of this transcript were reduced on average between 85 and 95% (depending on the flower stage and compared to WT plants) with the exception of ir-*hd20*-1 that showed WT levels of *HD20* expression in the closed-bud stage (CB; Figure 2b; univariate ANOVA, *F*_*14,44*_ = 26.64 *P* < 0.001 followed by a LSD *post-hoc* test *P* < 0.01). In corollas, the reduction in *HD20* mRNA levels was 87% in ir-*hd20*-1 and 97% in ir-*hd20*-2 lines (Figure 2b). The morphology and growth of ir-*hd20* plants were indistinguishable from those of WT (Figure 2a and Additional file 2). Flower morphology, flowering time and time of corolla opening were also similar between ir-*hd20* and WT plants (Figures 2c,d and Additional file 2). Consistent with *N. attenuata* plants silenced in *HD20* expression by VIGS [27], bolting time was delayed in ir-*hd20* plants compared to WT (Additional file 2).

### *HD20* positively regulates benzyl acetone emission from corollas

**Figure 3:**
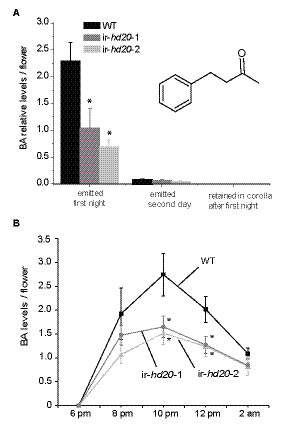
**Analysis of BA produced in corollas of*ir-hd20*and WT plants**. **(A)** The BA emitted from individual night flowers was trapped from 3 pm to 9 am and analyzed by GC-MS. The BA levels are expressed as the normalized peak areas to tetralin (internal standard; IS). Emitted BA was analyzed from both flowers that just opened their corollas (emitted first night) and from the same flowers but the day after (emitted second day). BA was also extracted from corolla tissue after the first night (retained in corollas after the first night). Asterisks denote significant differences; univariate ANOVA, *F*_*2,37*_ = 5.65 *P* < 0.01 followed by a LSD *post-hoc* test *P* < 0.01 for both lines versus WT; *n > 10*; bars denote ± SE. **(B)** Kinetic of BA emission from flowers at different times during corolla opening. Emitted BA was analyzed in real time with a zNOSE^TM^. Asteriks denote significant differences; univariate ANOVA, 10 pm: *F*_*2,12*_ = 4.18 *P* < 0.05, 12 am: *F*_*2,12*_ = 4.20 *P* < 0.05 followed by a LSD *post-hoc* test *P* < 0.05 for both lines versus WT; *n > 10*; bars denote ± SE).

Consistent with our previous observations [27], the emission of BA from corollas of ir-*hd20* plants was reduced by 60% to 70% compared to corollas of WT plants (Figure 3a; univariate ANOVA, *F*_*2,37*_ = 5.65 *P* < 0.01 followed by a LSD *post-hoc* test *P* < 0.01 for both lines versus WT). In this case, the emitted BA was trapped from individual flowers from 3 pm to 9 am (18 h trapping period). BA was the only volatile detected by GC-MS (gas chromatography–mass spectrometry) from flower headspace samples that showed differential accumulation in ir-*hd20* samples compared to WT. A second trapping period was also carried out during the second day after the corolla opening (from 3 pm to 9 am; 18 h trapping period). In this case, the levels of emitted BA were similar between WT and ir-*hd20* plants and were less than 5% of the BA levels emitted during the first night (Figure 3a). The analysis of BA levels retained in the corolla after the first night showed that these levels were 1,000 times lower than the amounts of emitted BA (Figure 3a). Analysis of BA emission in real time with a zNOSE^TM^ (Figure 3b; univariate ANOVA, 10 pm: *F*_*2,12*_ = 4.18 *P* < 0.05, 12 am: *F*_*2,12*_ = 4.20 *P* < 0.05 followed by a LSD *post-hoc* test *P* < 0.05 for both lines versus WT) showed that the amount of BA released by corollas of ir-*hd20* plants was significantly reduced compared to those of WT plants only after 8 pm.

### Changes in ABA levels correlate with changes in BA emission during corolla opening

**Figure 4:**
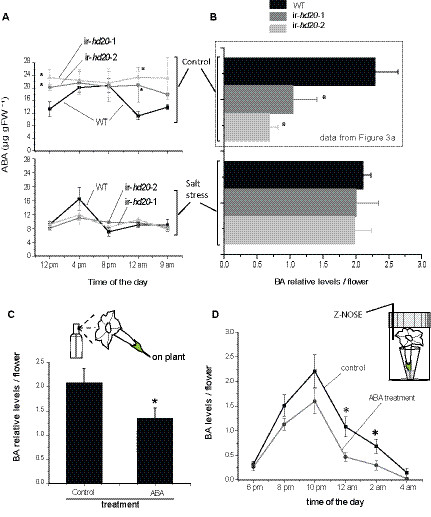
**Analysis of ABA levels in corolla and their effect on BA emission. (A)** ABA levels were quantified by LC-MS/MS in isolated corollas from flowers of WT and ir-*hd20* plants grown either under control or salt stress conditions. **(B)** BA was trapped from individual flowers from 3 pm to 9 am during the first night of corolla opening. WT and ir-*hd20* plants were grown either under control or salt stress conditions (BA levels in control conditions correspond to the data showed in Figure 3a and were included here for comparison). **(C)** Flowers from WT plants were sprayed either with 1 mL of solvent or with 1 mL a solution containing ABA (1 μg mL^-1^) at 8 pm. BA was trapped from individual flowers from 10 pm to 9 am and analyzed by GC-MS. BA levels are expressed as detailed in legend of Figure 3. **(D)** Flowers from WT plants were clipped off at the base of the pedicle and placed inside a microcentrifuge tube containing either 100 μL of water or 100 μL ABA (1 μg mL^-1^). Emitted BA was analyzed in real time with a zNOSE^TM^. Asterisks correspond to *P* < 0.05 (Student’s *t*-test; ir-*hd20 vs.* WT levels; *n* = 6; bars denote ± SE).

To begin to investigate the mechanisms underlying the reduced emission of BA from corollas of ir-*hd20* plants, we first profiled the accumulation of phytohormones in this tissue during the period of corolla opening. The levels of jasmonic acid (JA) and salicylic acid (SA) transiently increased during corolla opening to reach maximum levels at 8 pm and to decrease afterwards (Additional file 3). The levels of JA-Ile in corollas were on average 4 to 10 fold higher than the levels of JA (depending on the time) and they fluctuated on average between 0.5 and 1 μg gFW^-1^ (Additional file 3). The levels of JA, JA-Ile and SA were however not significantly different between WT and ir-*hd20* plants. In contrast, the levels of ABA differed between these two genotypes. In corollas of WT plants, the level of ABA started to increase at 12 pm and reached 20 μg gFW^-1^ at 4 pm (Figure 4a). The levels remained approximately constant until 8 pm and then started to decrease to reach 10 μg gFW^-1^ at 12 am (Figure 4a). This oscillation in ABA levels was not observed in corollas of ir-*hd20* plants, where ABA levels remained approximately constant at 20 to 24 μg gFW^-1^ during the time of corolla opening (Figure 4a; univariate ANOVA, 12 pm: *F*_*2,11*_ = 8.25 *P* < 0.01 followed by a LSD *post-hoc* test *P* < 0.01; 12 am: *F*_*2,11*_ = 5.39 *P* < 0.05 followed by a LSD *post-hoc* test *P* < 0.05 for both lines versus WT). Finally, the levels of ethylene emitted by the corolla were also quantified between 4 to 8:30 pm and 6 to 10:30 pm and they were similar between WT and ir-*hd20* plants (Additional file 3).

To investigate whether the changes in ABA levels quantified in corollas of ir-*hd20* plants were specific to this tissue and not the effect of altered ABA accumulation in the whole flower during development, phytohormone levels were quantified in whole flowers (corollas plus pistils, stamens, ovaries and nectaries) at progressive developmental stages. The results showed that accumulation of ABA in whole flowers was similar between WT and ir-*hd20* plants (Additional file 4) and hence the changes in ABA accumulation in corollas of ir**-***hd20* plants do not result from a general alteration of whole flower ABA levels.

To further investigate this association, *N. attenuata* WT and ir-*hd20* plants were grown under salt stress, a condition that maintained ABA in corollas at levels lower than 20 μg gFW^-1^ and almost completely abrogated the developmental fluctuation of this phytohormone in corollas of WT plants (Figure 4a). The salt stress did not affect BA emission from corollas of WT plants (compared to control growth conditions; Figure 4b). However, salt stress conditions recovered BA emission in ir-*hd20* plants and the levels of emitted BA were similar to those in WT plants (Figure 4b). From these results, we hypothesized that high levels of ABA in corollas (*i.e.* 20 μg gFW^-1^ or more) have a negative effect on BA emission and that the developmentally controlled 2-fold reduction in ABA levels in corollas of WT plants contributes to the emission of this volatile. To test this hypothesis, exogenous ABA was supplied to flowers of WT plants and the level of BA emitted from the corollas was quantified. Two different approaches were used: (1) flowers attached to the plant were sprayed with 1 mL of a solution containing 1 μg mL^-1^ ABA at 8 pm, and (2) clipped flowers were placed in scintillation vials and continuously fed through the pedicle with 100 μL of a solution containing 1 μg mL^-1^ ABA. The levels of ABA used in these experiments were in the range of endogenous ABA levels quantified in corollas (for spraying, we estimated a 90% loss of solution by measuring the run off). In the first approach, the emitted BA was trapped from 3 pm to 9 am (18 h) and analyzed by GC-MS (Figure 4c). In the second approach, the emitted BA was analyzed every two hours starting at 6 pm with a zNOSE^TM^ (Figure 4d). Corollas from WT plants sprayed with ABA emitted 30% less BA than control-treated flowers during the night (Figure 4c). Consistently, continuously pedicle-fed flowers emitted 25 to 50% lower levels of BA at 12 am and 2 am compared to control-treated flowers (Figure 4d).

In summary, these experiments indicated that ABA levels in corollas contributed either directly or indirectly to the release of BA. The negative effect of ABA on BA emission was however only significant after 10 pm, suggesting that the developmentally controlled reduction in ABA levels in corollas of WT plants contributes to the late phase of BA release. This conclusion is consistent with the reduction in ABA levels after 8 pm in corollas of WT plants (Figure 4a).

### Reduced levels of *HD20* in corollas have strong effects on gene expression

**Figure 5:**
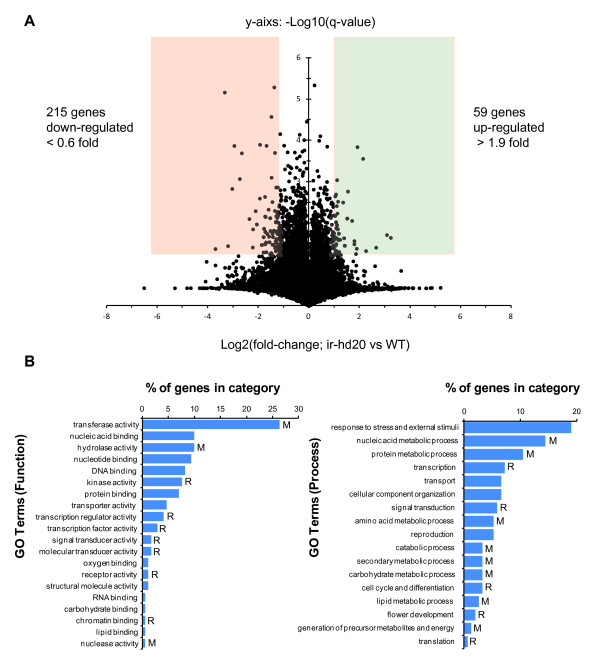
**Microarray analysis of gene expression in corollas of ir-*hd20*and WT plants.** Corollas from ir-*hd20* and WT flowers were collected at 12 pm and total RNA was extracted for analysis of gene expression using an Agilent Chip containing 43,533 *N. attenuata* probes. **(A)** Volcano plot showing the significant changes in gene expression in corollas of ir-*hd20* plants versus WT. **(B)** Gene Ontology (GO) analysis of the genes changing expression in corollas of ir-*hd20* plants. M: metabolism; R: regulatory activity.

To study the effect of *HD20* on the expression of genes in opening corollas, a *N. attenuata* Agilent custom-array containing 43,533 probes was used [28,29]. RNA was isolated from corollas of WT and ir-*hd20* plants at 12 pm (6 h before the start of BA emission) and used for microarray hybridization (see Materials and Methods for a detailed description of the method and data analysis). The results of the analysis showed that 215 genes were down- and 59 up-regulated (ir-*hd20 vs.* WT; 0.6 > FC (fold change) >1.9) significantly (q-value < 0.048; FDR 5.2%) in corollas of ir-*hd20* plants compared to WT (Additional file 5 and Figure 5a).

The genes affected in their expression were first categorized based on gene ontology (GO; process and function). Based on these two categories, between 44 and 37% of the annotated genes were involved in metabolic processes, respectively (Figure 5b, bars labeled with “M”). Analysis of GO categories (EC; Additional file 5), revealed that the most prevalent changes in gene expression occurred in enzymes involved in metabolic processes associated to aromatic compounds (Table 1). More specifically, genes involved in the metabolism of the aromatic amino acids tyrosine, tryptophan and phenylalanine (derivatives of the shikimate pathway) and the biosynthesis of phenylpropanoid derivatives were differentially expressed in corollas of ir-*hd20* plants compared to WT (Table 1). A putative *L*-amino-acid oxidase (EC:1.4.3.2) involved in the metabolism of tyrosine, tryptophan and phenylalanine and an unspecific monooxygenase (EC:1.14.14.1) involved in the metabolism of tryptophan were down-regulated in corollas of ir-*hd20* plants while the transcript levels of the rest of the genes involved in aromatic compound metabolism were up-regulated (Table 1). The second largest group of metabolic genes affected in their expression in corollas of ir-*hd20* plants was associated with carbohydrate metabolism (Table 1). In this case, several genes involved in sugar metabolism were down-regulated while two genes involved in cell wall biosynthesis were up-regulated. Core genes of the fatty acid and lipid biosynthesis pathways were also affected, and in this case they were up-regulated in corollas of ir-*hd20* plants (Table 1). Together, the changes in the expression of genes involved in aromatic compound metabolism and in sugar and lipid metabolism suggested that HD20 participates either directly or indirectly in the control of primary and secondary metabolism in corollas of *N. attenuata.*

**Table 1:** List of selected genes involved in biochemical pathways

**Metabolic pathway**	**Gene function**	**Gene ID**	FC*	**q-value**
**Aromatic compounds**				
Tryptophan metabolism	EC:1.14.14.1 - unspecific monooxygenase	Na_14909	0.6	0.0241
	EC:1.4.3.2 - L-amino-acid oxidase	Na_16251	0.6	0.0447
	EC:4.1.1.28 - aromatic-L-amino-acid decarboxylase	Na_32067	2.1	0.0080
Tyrosine metabolism	EC:1.4.3.2 - L-amino-acid oxidase	Na_16251	0.6	0.0447
	EC:4.1.1.28 - aromatic-L-amino-acid decarboxylase	Na_32067	2.1	0.0080
	EC:4.1.1.25 - tyrosine decarboxylase	Na_32067	2.1	0.0080
Phenylalanine metabolism	EC:1.4.3.2 - L-amino-acid oxidase	Na_16251	0.6	0.0447
	EC:4.1.1.28 - aromatic-L-amino-acid decarboxylase	Na_32067	2.1	0.0080
Biosynthesis of	EC:1.1.1.219 - dihydroflavonol 4-reductase	Na_06408	2.1	0.0058
phenylpropanoid derivatives	EC:2.3.1.74 - chalcone synthase	Na_02855	2.1	0.0241
	EC:1.14.11.19 - leucocyanidin oxygenase	Na_12114	2.2	0.0019
**Carbohydrate metabolism**				
Starch and sucrose	EC:2.4.1.21 - starch synthase	Na_41693	0.3	0.0223
metabolism	EC:2.7.1.4 - fructokinase	Na_26865	0.5	0.0226
	EC:2.4.1.15 - alpha,alpha-trehalose-phosphate synthase (UDP-forming)	Na_27299	0.5	0.0266
	EC:3.1.3.12 - trehalose-phosphatase	Na_27299	0.5	0.0266
Cell wall biosynthesis	EC:2.4.1.12 - cellulose synthase (UDP-forming)	Na_10287	1.9	0.0147
	EC:2.4.1.12 - cellulose synthase (UDP-forming)	Na_28221	2.0	0.0260
**Lipid metabolism**				
Glycerophospholipid metabolism	EC:2.1.1.103 - phosphoethanolamine N-methyltransferase	Na_18104	1.9	0.0299
	EC:3.1.4.46 - glycerophosphodiester phosphodiesterase	Na_34149	2.4	0.0117
Fatty acid biosynthesis	EC:2.3.1.86 - fatty-acyl-CoA synthase	Na_20395	1.9	0.0351

* FC: fold-change (ir-*hd20 vs.* WT).

GO categorization also showed that 20% of the genes affected in their expression were involved in regulatory processes (Figure 5b, bars labeled with “R”). Among these genes, the most prevalent group was associated to the regulation of gene expression and it included two WRKY transcription factors and two chromatin-remodeling factors (Table 2). The expression of several genes with protein kinase activity (including receptors such as ETR1) was also affected (Table 2). Among the down-regulated receptor proteins was *CORONATINE INSENSITIVE 1* (*COI1*), previously associated with developmental processes in flowers [30-32].

**Table 2:** List of selected genes involved in regulatory processes

**Gene description**	FC*	**q-value**	**Gene ID**
**Gene expression**			
GBF S pro-rich region-interacting factor 1	0.3	0.0030	Na_05287
WRKY transcription factor	0.3	0.0120	Na_30800
Fungal-specific transcription factor domain protein	0.4	0.0140	Na_31163
Histone acetyltransferase complex component	0.4	0.0009	Na_15638
RWP-RK domain-containing protein	0.5	0.0479	Na_20983
Chromatin remodeling complex subunit	0.5	0.0395	Na_16219
WRKY transcription factor 29	0.5	0.0420	Na_36997
RNA polymerase sigma factor	0.6	0.0222	Na_13263
Paired amphipathic helix protein SIN3-like 2	0.6	0.0415	Na_13033
GRAS transcription factor	4.8	0.0360	Na_40201
**Protein kinases and phosphatases**			
Casein kinase	0.1	0.0051	Na_35212
Ser-Thr protein phosphatase	0.3	0.0158	Na_21633
Protein phosphatase 2 C	0.6	0.0436	Na_30444
TCTR2 protein kinase	0.4	0.0121	Na_36065
**Receptor activity**			
Ethylene receptor 1 (ETR1)	0.4	0.0144	Na_24848
S-locus lectin protein kinase	0.5	0.0147	Na_14354
Coronatine insensitive 1 (COI1)	0.6	0.0283	Na_04958
LRR receptor-like kinase	0.6	0.0388	Na_29192
**Other regulatory activities**			
circadian clock-associated FKF1	0.3	0.0002	Na_17932

* FC: fold-change (ir-*hd20 vs.* WT).

**Figure 6:**
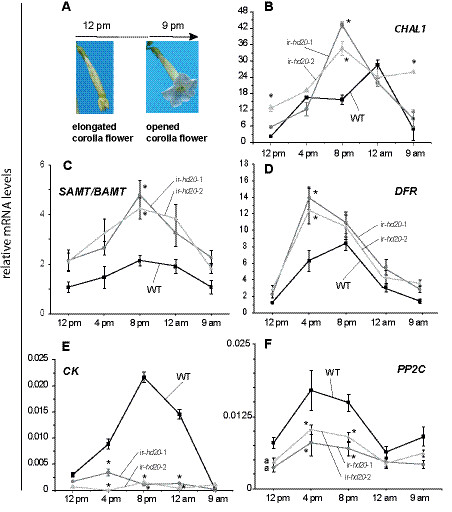
**Analysis of gene expression during corolla opening by qPCR.** Total RNA was extracted from isolated corollas of ir-*hd20* and WT plants at different times during corolla opening. Total RNA was reversed transcribed and the mRNA levels for the different genes were quantified by qPCR. Transcript levels are expressed as relative units to the levels of the reference *EF1A* mRNA as calculated by the ΔCT method. Asterisks denote significant differences; univariate ANOVA *F*_*2,8*_ and *P* < 0.05 followed by a LSD *post-hoc* test *P* < 0.05 for both lines versus WT; *n* = 3; bars denote ± SE. **(A)** Schematic representation of corolla opening. **(B)***CHAL1* mRNA levels. **(C)***SAMT/BAMT* mRNA levels. **(D)***DFR* mRNA levels. **(E)***CK* mRNA levels. **(F)***PP2C* mRNA levels.

To further assess the changes in gene expression in corollas of ir-*hd20* plants, the kinetics of expression of a selected group of genes identified by microarray analysis were evaluated by qPCR during the time of corolla opening (Figure 6a). Three of the selected genes are involved in aromatic compound metabolism, namely *Chalcone Synthase 1* (*CHAL1*), *S-adenosyl-L-methionine:Benzoic acid/Salicylic acid Carboxyl Methyltransferase* (*SAMT/BAMT*) and *Dihydroflavonol 4-Reductase* (*DFR*)(Table 1). *CHAL1* has been previously associated with BA production in *N. attenuata* plants [2]. The accumulation of these three transcripts was induced in corollas of WT plants during the time of corolla opening to reach maximum levels between 4 and 8 pm (Figure 6b,c,d). Consistent with the microarray data, the levels of *CHAL1SAMT/BAMT* and *DFR* mRNAs were increased in corollas of ir-*hd20* plants compared to WT (Figure 6b,c,d; univariate ANOVA, *F*_*2,8*_ and *P* < 0.05 followed by a LSD *post-hoc* test *P* < 0.05 for both lines versus WT).

The expression of two regulatory genes was also analyzed, namely *Casein Kinase* (*CK*; the most strongly down-regulated gene; Additional file 5 and Table 2) and *Protein Phosphatase 2 C* (*PP2C*). The expression of *CK* was induced several fold during corolla opening in WT plants and reached a maximum at 8 pm (Figure 6e). In contrast, in corollas of ir-*hd20* plants, the levels of this transcript remained largely non-induced (Figure 6e; univariate ANOVA, *F*_*2,8*_ and *P* < 0.05 followed by a LSD *post-hoc* test *P* < 0.05 for both lines versus WT). Finally, the levels of *PP2C* mRNA were induced by 2-fold during corolla opening in WT plants and the levels remained approximately 2-fold lower in corollas of ir-*hd20* plants (Figure 6f; univariate ANOVA, *F*_*2,8*_ and *P* < 0.05 followed by a LSD *post-hoc* test *P* < 0.05 for both lines versus WT).

## Discussion

### Changes in ABA levels in opening corollas are associated with BA emission

The expression of *HD20* in flowers and corollas was consistent with the participation of this transcription factor in the process of BA emission. The levels of *HD20* mRNA were highest in corollas and they increased during the time of corolla opening (Figure 1). The timing of induction correlated with the timing of BA emission which starts in the afternoon and peaks between 9 and 10 pm (Figure 3b). Several HD-Zip type-I transcription factors in different plants species are either highly expressed in flowers or have been associated with mechanisms regulating organ development. For example, LeHB-1 from tomato (*Solanum lycopersicum*) regulates floral organogenesis [24] and the Arabidopsis *HB12HB7HB6* and *HB1* are highly expressed in flowers [19,20]. Based on phylogenetic analysis, the Arabidopsis *HB12* and *HB7* genes group together with *HD20* in subgroup Ic [27,33].

Consistent with our previous study in which the expression of *HD20* was reduced by VIGS in *N. attenuata* plants [27], plants with stably reduced expression of *HD20* were also reduced in the levels of BA released from corollas (Figure 3). Analysis of BA emission during the first and second nights showed that flowers from ir-*hd20* plants produced less BA with no delay in its emission (Figure 3a). Moreover, analysis of the dynamics of BA emission showed that corollas of ir-*hd20* plants released significantly smaller BA amounts after 8 pm, indicating that reduced *HD20* expression affected primarily the late phase of BA emission (Figure 3b).

In WT plants, *HD20* mRNA levels were detected throughout the time of corolla opening (*i.e.* from 12 pm to 12 am) and peaked at 8 pm (Figure 1b). ABA levels increased in corollas of WT plants from 12 pm to 4 pm and decrease from 8 pm to 9 am (Figure 4a). In corollas of ir-*hd20* plants, this fluctuation in ABA levels was not observed (Figure 4a) however when ABA levels in corollas were reduced by salt stress treatment, the emission of BA was recovered to WT levels (Figure 4b). Moreover, feeding experiments with exogenous ABA decreased BA emission at the late phase of the process (*i.e.* after 10 pm; Figures 4c and d). Based on these observations, we concluded that there was a direct association between the decline of ABA levels in corolla and the late phase of BA emission. Thus, the results suggested that HD20 contributes partially to BA emission by directly or indirectly affecting ABA levels. How HD20 affects ABA levels in corollas is at present unknown. Genome-wide gene expression analysis performed with corolla tissue harvested at 12 pm (6 h before the start of BA emission) did not detect genes involved in ABA biosynthesis as differentially expressed. At present, however, it is unknown how the corolla controls ABA levels, this could be the result of *de novo* biosynthesis, import from adjacent tissue, metabolism (*i.e.* turnover) or a combination of these processes. Although at 12 pm, 2-fold higher levels of ABA were detected in corollas of ir-*hd20* plants compared to WT (Figure 4a), only one gene homolog to *Zea mays* SnRK2 (known to be regulated by ABA [34]) was identified as differentially expressed (supplementary file 5). Little is known about the regulation of gene expression by ABA in corollas and ABA is known to affect multiple cellular processes (*e.g.*, opening of ion channels, protein phosphorylation) in addition to gene expression [35]. Moreover, the direct target genes of HD20 are thus far unknown in *N. attenuata*. The mechanisms underlying the reduction in BA emission mediated by HD20 and ABA are probably very complex and additional experimentation will be required to disentangle this complexity.

### Changes in metabolic gene expression induced by HD20 are associated with BA emission

*Chalcone Synthase 1* (*CHAL1*) is so far the only gene that has been involved in the biosynthesis of BA in *N. attenuata* plants [2]. In other words, the biosynthesis pathway for this floral volatile is largely unknown. Chalcone synthases catalyze the first step of flavonoid biosynthesis using malonyl-coenzyme A (CoA) and 4-coumaroyl-CoA to produce naringenin chalcone [36]. 4-coumaroyl-CoA is supplied by the phenylpropanoid pathway which utilizes phenylalanine produced by the shikimate pathway in the plastid. Tyrosine and tryptophan are also produced by this biosynthesis pathway. Naringenin chalcone can be further modified to form flavonoids and dihydroflavonol 4-reductase (DFR) is the first committed enzyme of anthocyanin biosynthesis in the flavonoid pathway [36]. Benzoids are also derived from the phenylpropanoid or shikimate pathways and enzymes such as S-adenosyl-L-methionine:benzoic acid/Salicylic acid carboxyl methyltransferase (BAMT/SAMT) synthesize methyl esters (*e.g.*, methyl benzoate and methyl salicylate) which are constituents of floral scents of many plant species [1]. In *Petunia hybrida*, where benzenoids determine the floral scent, three transcription factors from the R2R3-type MYB family have been identified that regulate floral scent production. *ODORANT1* (*ODO1*), *EMISSION OF BENZENOIDS II* (*EOBII*) and *PhMYB4* affect the expression of several biosynthetic floral scent-related genes encoding enzymes from the phenylpropanoid and shikimate pathways [5,6,37].

The analysis of changes in gene expression in opening corollas of ir-*hd20* plants showed that the most prevalent group of genes with altered expression corresponded to those involved in aromatic compound metabolism and derivatives of the phenylpropanoid biosynthesis pathway. Importantly, no candidate genes involved in BA biosynthesis could be identified, however, as the genes involved in this process are largely unknown and a large fraction of the genes differentially expressed in corollas of ir-*hd20* are of undescribed function (supplementary file 5), it is plausible that among these genes some involved in BA biosynthesis are present.

The induction of expression of genes involved in aromatic compound metabolism and derivatives of the phenylpropanoid biosynthesis pathway during corolla opening (*e.g.*, *CHAL1*, *SAMT/BAMT*, *DFR*) correlated positively with the emission of BA and with the turning of the corolla limb from green to white. Thus, these observations were consistent with the altered levels of BA production in ir-*hd20* plants and suggested that reduced emission of this floral volatile could be the result of altered gene expression in BA biosynthesis genes. Paradoxically, however, in addition to *CHAL1*, several other genes involved in different steps of aromatic compound biosynthesis or metabolism were up-regulated in corollas of ir-*hd20* plants (Table 1). Because several genes involved in carbohydrate metabolism were down-regulated in corollas of these plants (Table 1), one plausible explanation for the reduced levels of BA emission in ir-*hd20* plants is that reduced carbon fluxes through the shikimate and phenylpropanoid pathways limit BA production. If this is the case, the increased levels of chalcone synthase and other phenylpropanoid biosynthesis genes observed in ir-*hd20* plants may reflect a compensatory mechanism for reduced supply of carbon precursors. A detailed study of the metabolic fluxes of the shikimate and phenylpropanoid pathways in ir-*hd20* plants will be necessary to test this hypothesis in the future.

In addition to metabolic genes, multiple regulatory factors also showed altered expression in opening corollas of ir-*hd20* plants (Table 2). Among the genes in the group of protein kinases and phosphatases were *N. attenuata* homologues of casein kinase and PP2C (Table 2). Casein kinases (CKs) are ubiquitous Ser/Thr kinases that play critical roles in all higher organisms including plants. For example, the Arabidopsis CK2 plays important roles in light signaling, circadian rhythms, hormone responses, cell cycle control and flowering time [6,38,39]. The strong down-regulation of casein kinase suggests that this protein kinase may be important in regulating directly or indirectly BA emissions by HD20-dependent mechanisms. Interestingly, similar to HB12 and HB7 in Arabidopsis [33], the carboxyl terminus of HD20 presents three putative phosphorylation sites (data not shown), suggesting that phosphorylation/dephosphorylation mechanisms can be important for the regulation of its activity.

In connection with ABA signaling, a homologue of PP2C was down-regulated approximately two-fold in opening corollas of ir-*hd20* plants (Table 2 and Figure 6f). PPC2s such as ABI1 and ABI2 (Abscisic Acid Insensitive 1 and 2, respectively) are rapidly inactivated upon the binding of ABA to RCARs/PYR1/PYLs [5,40,41]. Inactivation of PP2Cs triggers the activation of SNF1-type kinases that initiate ABA-dependent responses such as the activation of gene expression and the regulation of ion channels [5,40,41]. As their names indicate, mutations in *ABI1* and *ABI2* genes make plants insensitive to ABA [42]. Functional *ABI1* and *ABI2* alleles are necessary for the induction of the Arabidopsis *HB12* and *HB7* by ABA [20]. Thus, in addition to the de-regulation of ABA levels in corollas of ir-*hd20* plants, sensitivity to this phytohormone could also be affected in corollas of these plants.

Other interesting genes with receptor activity that changed expression in opening corollas of ir-*hd20* compared to WT plants were *Ethylene receptor 1* (*ETR1*), *Coronatine insensitive 1* (*COI1*) and the circadian clock-associated *Flavin-binding Kelch repeat F-box 1* (*FKF1*) gene [43]. Ethylene and JA-Ile are important phytohormones for flower development. For example, plants deficient in the expression of *COI1* are sterile [31,32,44] and plants expressing a constitutively active allele of *ETR1* have delayed corolla senescence [45,46]. Although JA, JA-Ile and ethylene levels were not affected in corollas of ir-*hd20* plants, changes in the sensitivity to these phytohormones may also participate in the regulation of BA emission.

## Conclusion

In summary, from the results presented in this study it is clear that multiple metabolic pathways were affected when corollas were reduced in *HD20* expression, pointing to the importance of this HD-Zip transcription factor in the regulation of biochemical processes in this tissue. Importantly, reduced expression of *HD20* did not cause morphological and developmental changes in flowers, indicating that the main function of this transcription factor in this organ relates to the regulation of metabolism rather than development. The mechanisms underlying the regulation of BA levels by HD20 are clearly complex and they most likely involve a complex network of factors and ABA signaling. As the sequences of the promoter regions of *N. attenuata* genes become available with the sequencing of this plant’s genome, the analysis of the presence of the universally conserved HD-Zip type I binding element (CAAT(A/T)ATTG)[14,15] in genes affected in their expression in ir-*hd20* plants will facilitate the discovery of direct target genes of HD20.

## Methods

### Plant growth and treatments

Seeds of *N. attenuata* plants were germinated on agar plates containing Gamborg's B5 medium as previously described [47]. Plates were maintained in a growth chamber (Snijders Scientific, Tilburg, Netherlands) at 26°C/16 h (155 μmol s^-1^ m^-2^ light), 24°C/8 h dark for 10 days. Ten-day old seedlings were transferred to TEKU pots (Pöppelmann GmbH & Co. KG, Lohne, Germany) with Klasmann plug soil (Klasmann-Deilmann GmbH, Geesten, Germany). After 10 days, seedlings were transferred to soil in 2 L pots and grown in the glasshouse under high-pressure sodium lamps (200-300 μmol s^−1^ m^−2^) with a day/night ratio of 16 h (26–28°C)/8 h (22–24°C) and 45–55% humidity. Plants were grown in pairs (one WT and one ir-*hd20* per 2-L pot).

For salt stress treatments, elongated *N. attenuata* plants were watered daily under a regime of: 2 days with an aqueous solution of 75 mM NaCl, 3 days with 150 mM NaCl and 4 days with 300 mM NaCl. The plants were left to soak the saline water for 1 h and the excess of liquid was removed from the trays. The saline water volume was 0.6 L per tray and 5 plant pots were placed per tray.

For exogenous ABA application, a solution of 1 μg mL^-1^ ABA in 0.02% (v/v) Tween-20/water was sprayed (1 mL per flower) on corollas at 8 pm with a perfume dispenser. The control solution was 0.02% (v/v) Tween-20/water. Flowers were immediately enclosed in volatile traps for BA analysis by GC-MS (see below). For pedicle feeding of ABA, individual clipped flowers were placed inside 1.7 mL microcentrifuge tubes (with their caps removed) containing 100 μL of either water or ABA (1 μg mL^-1^) dissolved in water. The microcentrifuge tubes carrying the flowers were placed inside glass scintillation vials and the vials were screw-capped. BA levels were determined with a zNOSE^TM^ (Electronic Sensor Technology, Newbury Park, CA) every 2 h (see below), from 6 pm to 4 am. These experiments were performed with 6 biological replicates per genotype.

### Generation of transgenic ir-*hd20* lines

Transgenic *N. attenuata* (ir-*hd20*) plants reduced in the expression of *HD20* were generated via elongated hypocotyl *Agrobacterium*-mediated transformation and seedling regeneration as previously described [47]. The binary vector used for plant transformation was pSOL8 [48] engineered to carry a 329 bp fragment of the *HD20* mRNA corresponding to nucleotides 529 to 857 (GenBank accession: HM107874) and subcloned in inverted repeat orientation to generate pSOL8HD20. T_1_ transformed plants were analyzed by quantification of ABA levels (see below) and *HD20* mRNA levels (see below) in leaves during water stress(*i.e.* by withholding irrigation for 5 days) and for T-DNA single insertion by Southern blot hybridization (see below). Segregation analysis of hygromycin resistance in T_2_ seedlings was performed on agar plates supplemented with hygromycin (0.025 mg mL^-1^). Two lines, ir-*hd20* A-09-408 (ir-*hd20* line 1) and A-09-411 (ir-*hd20* line 2) had the lowest levels of ABA and *HD20* mRNA accumulation at 5 days after withholding water and had a single T-DNA insertion in their genomes. These lines were used for all experiments. Southern blot analysis was performed as previously described [29].

### Analysis of phytohormones

For analysis of JA, JA-Ile, SA and ABA, 0.2 g of frozen tissue were homogenized to a fine powder in the presence of liquid nitrogen. One mL of ethylacetate spiked with 200 ng [²H_2_JA and 40 ng ^13^ C_6_JA-Ile, ^2^ H_6_ABA, ^2^ H_4_SA was added to the samples and after vortexing the samples were centrifuged for 15 min at 13,200 g (4°C). The upper organic phase was transferred into a fresh tube and the leaf material was re-extracted with 0.5 mL ethylacetate. The organic phases were pooled and evaporated to dryness. The dry residue was reconstituted in 0.4 mL of 70/30 (v/v) methanol/water for analysis with an LC-ESI-MS/MS instrument (Varian 1200 Triple-Quadrupole-LC-MS system; Varian, Palo Alto, CA). Ten μL of the sample were injected in a ProntoSIL® column (C18-ace-EPS, 50 x 2 mm, 5 μm, 120 Å, Bischoff, Leonberg, Germany) connected to a pre-column (C18, 4 x 2 mm, Phenomenex, Torrance, CA). As mobile phases 0.05% formic acid in water (solvent A) and methanol (solvent B) were used in a gradient mode with the following conditions: time/concentration (min/%) for B: 0.0/15; 2.5/15; 4.5/98; 10.5/98; 12.0/15; 15.0/15; time/flow (min/mL): 0.0/0.4; 1.5/0.2; 1.5/0.2; 10.5/0.4; 15.0/0.4. Compounds were detected in the ESI negative mode and multiple reaction monitoring (MRM) according to published parameters [49].

### Ethylene (ET) measurements

ET levels were analyzed by photoacoustic spectrometry (INVIVO; https://www.invivo-gmbh.de) as previously described [50]. Corollas were excised from antherectomized flowers at different times (Additional file 3) and immediately placed in 100 mL sealed-glass vessels (four corollas per vessel) for 4.5 h before ET quantification. Three biological replicates per time and per genotype were performed. Antherectomization was performed in the morning before pollen release to avoid the production of ET induced by fertilization.

### Quantification of benzyl acetone (BA) emission from flowers

Flower volatiles were collected by enclosing individual night flowers in plastic cups connected to Super-Q filter traps (ARS, Philadelphia, PA) under an air flow of 30 mL min^-1^. Volatiles were collected from 3 pm to 9 am. Filter traps were spiked with 400 ng of tetraline as an internal standard (IS) and eluted with 250 μL of dichloromethane. Volatile analysis was performed by GC-MS with a CP-4000 GC instrument (Varian, Palo Alto, CA) on a DB-5 column (Agilent, Waldbronn, Germany). BA was identified by comparing retention times and mass spectra with a commercial BA standard (Sigma, Taufkirchen, Germany). Peak areas were integrated and normalized to the area of the IS.

BA was also analyzed by the zNOSE^TM^ as previously described [51]. In this case, individual flowers were placed in scintillation vials and the vials were left capped. Every 2 h (starting at 6 pm; Figure 4d) the needle of the zNOSE^TM^ was introduced into the vials to determine BA levels in the headspace. The vials were immediately capped again. The intensity of the peak area corresponding to BA was used to quantify BA levels per flower.

### Analysis of gene expression by qPCR

Total RNA was extracted from different tissues by the TRIzol® reagent (Invitrogen) and DNase-I treated (Fermentas, St. Leon-Rot, Germany) according to commercial instructions. Five μg of total RNA were reverse transcribed using oligo(dT)18 and SuperScript reverse transcriptase II (Invitrogen) according to commercial instructions. Quantitative real-time PCR (qPCR) was performed with the Mx3005P Multiplex qPCR system (Stratagene, La Jolla, CA) and the qPCR Core kit for SYBR® Green I (Eurogentec, Liege, Belgium) using gene specific primers (Additional file 6). Quantification of *HD20* mRNA levels was performed by normalization with the Elongation Factor 1A (*EF1A*) mRNA according to the ΔCt method [52]. All the reactions were performed with at least three biological replicates.

### Microarray analysis

Corollas from ir-*hd20* and WT plants were collected at 12 pm (a total of three independent samples (biological replicates) were used per genotype and each sample was composed of three pooled corollas). Total RNA was extracted from corollas based on a previously described method [53] and its quality was checked by spectrophotometry (NanoDrop, Wilmington, DE). Genomic DNA was removed by DNAse treatment following commercial instructions (Turbo DNase; Ambion, Europe), RNA was cleaned up with RNeasy MinElute columns (Qiagen, Hilden, Germany) and the RNA quality was checked with the RNA 6000 n kit (Agilent, Santa Clara, CA) using an Agilent 2100 Bioanalyzer. Total RNA was used to generate labeled cRNA with the Quick Amp labeling kit (Agilent) and the yield was determined spectrophotometrically (NanoDrop). Labeled cRNA was hybridized using the gene expression hybridization kit (Agilent) following commercial instructions onto 44 K custom-designed 60-mer *N. attenuata* Agilent microarray containing 43,533 sequences (see accession numbers)[28,29]. Microarrays were hybridized overnight at 65°C and slides were washed with the Gene Expression Wash Buffer kit (Agilent) as outlined in the One-Color Microarray-Based Gene Expression Analysis manual (Agilent). Three biological replicates were used per treatment with a total of six arrays. Arrays were scanned with an Agilent G2565BA scanner and image data was acquired with the Agilent Scan Control software (version A.7.0.1 for the B scanner). Data was extracted using the Agilent Feature Extraction software (version 9.5) and analyzed with the SAM (Significance Analysis of Microarrays) software [54]. The q-values for each gene corresponded to a computed false discovery rate (FDR) of 5.2%. Significant changes in gene expression were considered when the fold change (FC; ir-*hd20* versus WT) were equal or greater than 1.9 or equal or smaller than 0.6 (with q-values lower than 0.048; according to the FDR value calculated by SAM). Gene Ontology (GO) and enzyme code (EC) classification and sequence analysis was performed with the Blast2GO software [55].

### Statistical analysis

Statistics were calculated using the SPSS software version 17.0. The data was subjected either to one-way analysis of variance (ANOVA; and means were compared by the lowest standard deviation (LSD) test) or to Student’s *t*-test as indicated in the text. The number of replicates (*n*) used in each experiment are detailed in the figure’s captions.

### Accession numbers

Sequence data from this article can be found under the following accession numbers: HD20 [GenBank:HM107874], CHAL1 [GenBank:EU503226], FDR [GenBank:JQ028693], PP2C [GenBank:JQ028694], SAMT/BAMT [GenBank: JQ028692], Agilent Chip platform [NCBI GEO:GPL13527], and microarray data [NCBI GEO: GSE33682].

## Authors' contributions

DAR, BR and GB carried out the experiments and analyzed the data. ITB and RLC participated in the design of the study and edited the manuscript. GB conceived of the study and participated in its design and coordination. DAR and GB drafted the manuscript. All authors read and approved the final manuscript.

## Supplementary Material

Additional file 1 Southern blot analysis of ir-*hd20* plants. Click here for file

Additional file 2 Morphological and developmental characterization of ir-*hd20* plants. Click here for file

Additional file 3 Quantification of phytohormone levels in corollas of ir-*hd20* and WT plants during corolla opening. Click here for file

Additional file 4 Analysis of phytohormones in whole flowers of ir-*hd20* and WT plants at different developmental stages. Click here for file

Additional file 5 List of genes changing expression in corollas of ir-*hd20* plants compared to WT plants. Click here for file

Additional file 6 List of primers for qPCR analysis. Click here for file
