# Supplementary material for: RNAi-mediated silencing of the HD-Zip gene HD20 in Nicotiana attenuata affects benzyl acetone emission from corollas via ABA levels and the expression of metabolic genes
Source: BMC Plant Biol. 2012 May 1;12:60. doi: 10.1186/1471-2229-12-60 (PMC3413612; doi:10.1186/1471-2229-12-60)
Supplement: Additional file 1 — Southern blot analysis of ir-hd20 plants. [file 1471-2229-12-60-S1.pdf]

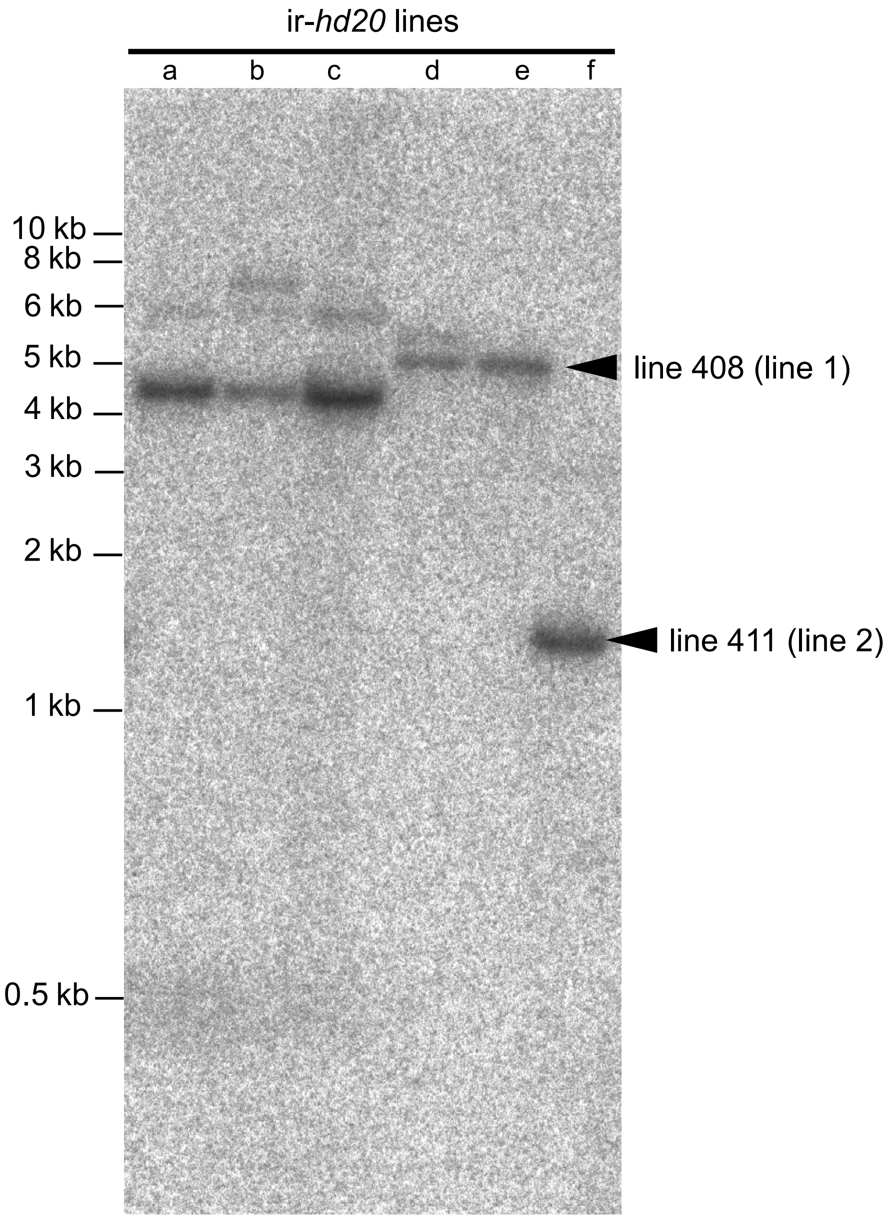

Figure S1. Southern blot analysis of ir-*hd20* plants.

Genomic DNA from six independent ir-*hd20* lines (a to f) and WT (not shown) was digested with EcoRV and resolved by agarose gel electrophoresis. A ^{32}P-labeled PCR product corresponding to the hygromycin resistance gene (*nptII*) was used as a probe. The arrow heads point to individual T-DNA insertions in the lines 408 (ir-*hd20*-1) and 411 (ir-*hd20*-2) used in this study.
