# Supplementary material for: RNAi-mediated silencing of the HD-Zip gene HD20 in Nicotiana attenuata affects benzyl acetone emission from corollas via ABA levels and the expression of metabolic genes
Source: BMC Plant Biol. 2012 May 1;12:60. doi: 10.1186/1471-2229-12-60 (PMC3413612; doi:10.1186/1471-2229-12-60)
Supplement: Additional file 2 — Morphological and developmental characterization of ir-hd20 plants. [file 1471-2229-12-60-S2.pdf]

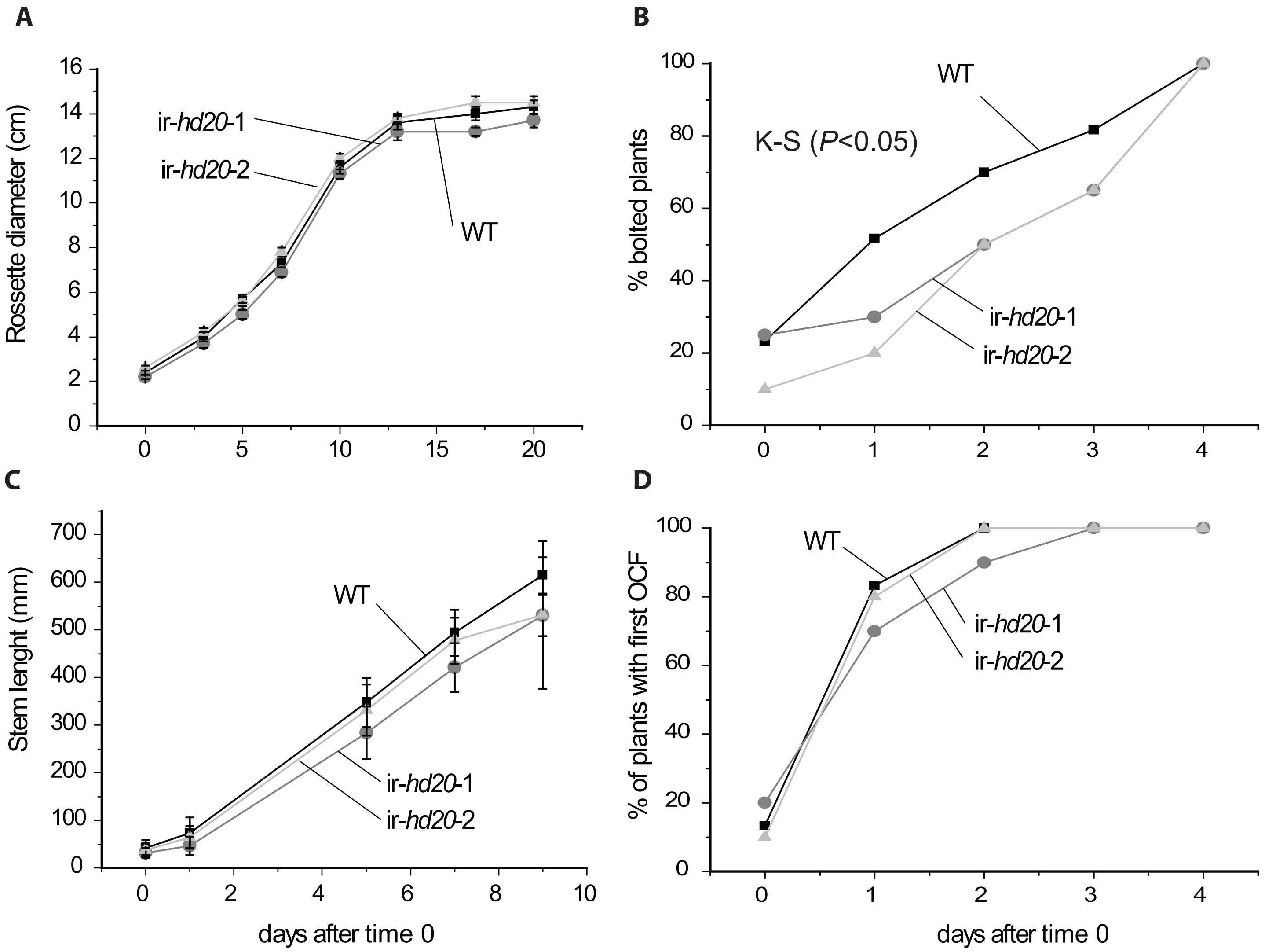

Figure S2. Morphological and developmental characterization of ir-hd20 plants

WT and ir-*hd20* *N. attenuata* plants were grown in the glasshouse under the conditions described in the Materials and Methods section. **(A)** Rosette diameter. The rosette diameter was defined as the distance between the tips of the two largest leaves (*n*=20 plants per genotype). Day 0 (D0) was defined in this case as the first day of diameter measurements. **(B)** Percentage (%) of bolted plants. Bolted plants were defined as plants showing the emerging inflorescence meristem. Day 0 in this case refers to the day in which the first plant bolted (two-sample Kolmogorov-Smirnov (K-S) test; *P*<10-4; *n*=20 plant per genotype). **(C)** Rate of stalk elongation. Day 0 in this case refers to the first day of stalk length measurements (*n*=20 plants per genotype). **(D)** Percentage (%) of plants that had at least one opened corolla flower (OCF). Day 0 in this case refers to the day in which the first plant showed an OCF (*n*=20 plants per genotype).
