# Supplementary material for: RNAi-mediated silencing of the HD-Zip gene HD20 in Nicotiana attenuata affects benzyl acetone emission from corollas via ABA levels and the expression of metabolic genes
Source: BMC Plant Biol. 2012 May 1;12:60. doi: 10.1186/1471-2229-12-60 (PMC3413612; doi:10.1186/1471-2229-12-60)
Supplement: Additional file 3 — Quantification of phytohormone levels in corollas of ir-hd20 and WT plants during corolla opening. [file 1471-2229-12-60-S3.pdf]

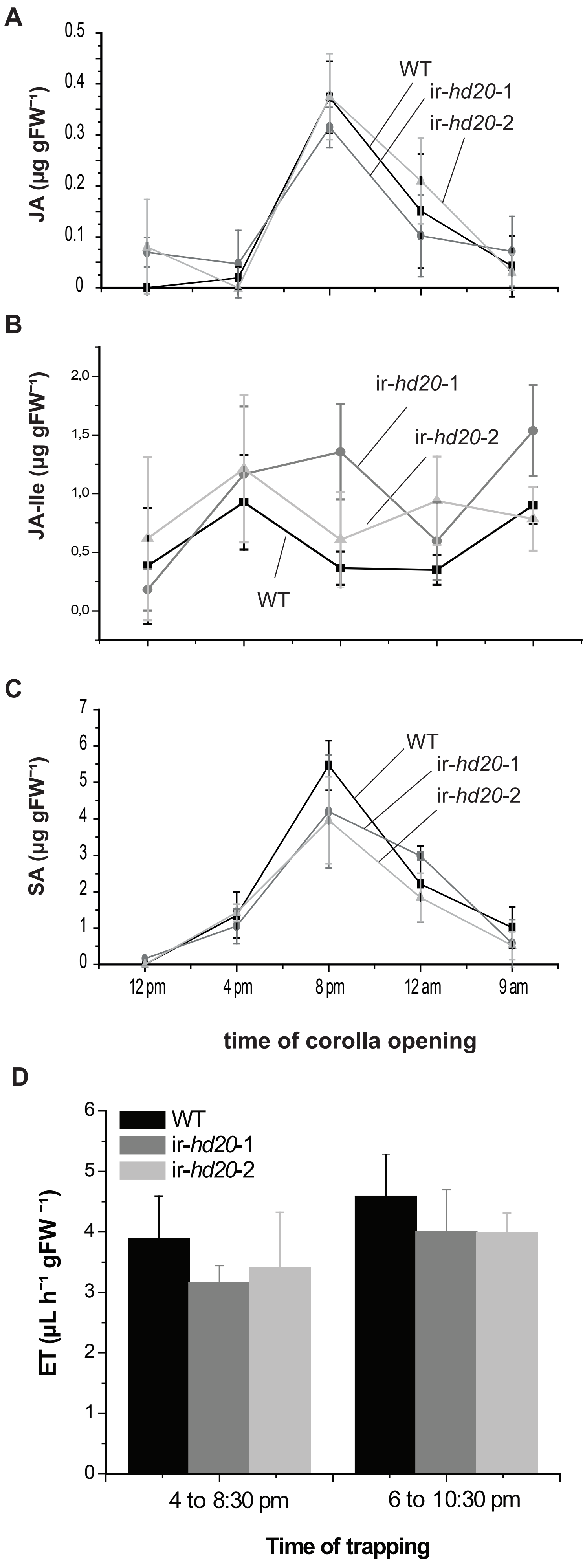

Figure S3. Quantification of phytohormone levels in corollas of ir-hd20 and WT plants during corolla opening.

Phytohormones were extracted from isolated corollas at different times of corolla opening and analyzed by LC-MS/MS. **(A)** JA levels. **(B)** JA-Ile levels. **(C)** SA levels. (n=4; bars denote ± SE). **(D)** Isolated corollas from emasculated ir-hd20 and WT flowers were collected at 4 and 6 pm and placed in 100 mL glass containers for 4.5 h for quantification of ethylene levels as described in the Materials and Methods section (four corollas were pooled per sample and a total of four samples per genotype were used).
