# Supplementary material for: RNAi-mediated silencing of the HD-Zip gene HD20 in Nicotiana attenuata affects benzyl acetone emission from corollas via ABA levels and the expression of metabolic genes
Source: BMC Plant Biol. 2012 May 1;12:60. doi: 10.1186/1471-2229-12-60 (PMC3413612; doi:10.1186/1471-2229-12-60)
Supplement: Additional file 4 — Analysis of phytohormones in whole flowers of ir-hd20 and WT plants at different developmental stages. [file 1471-2229-12-60-S4.pdf]

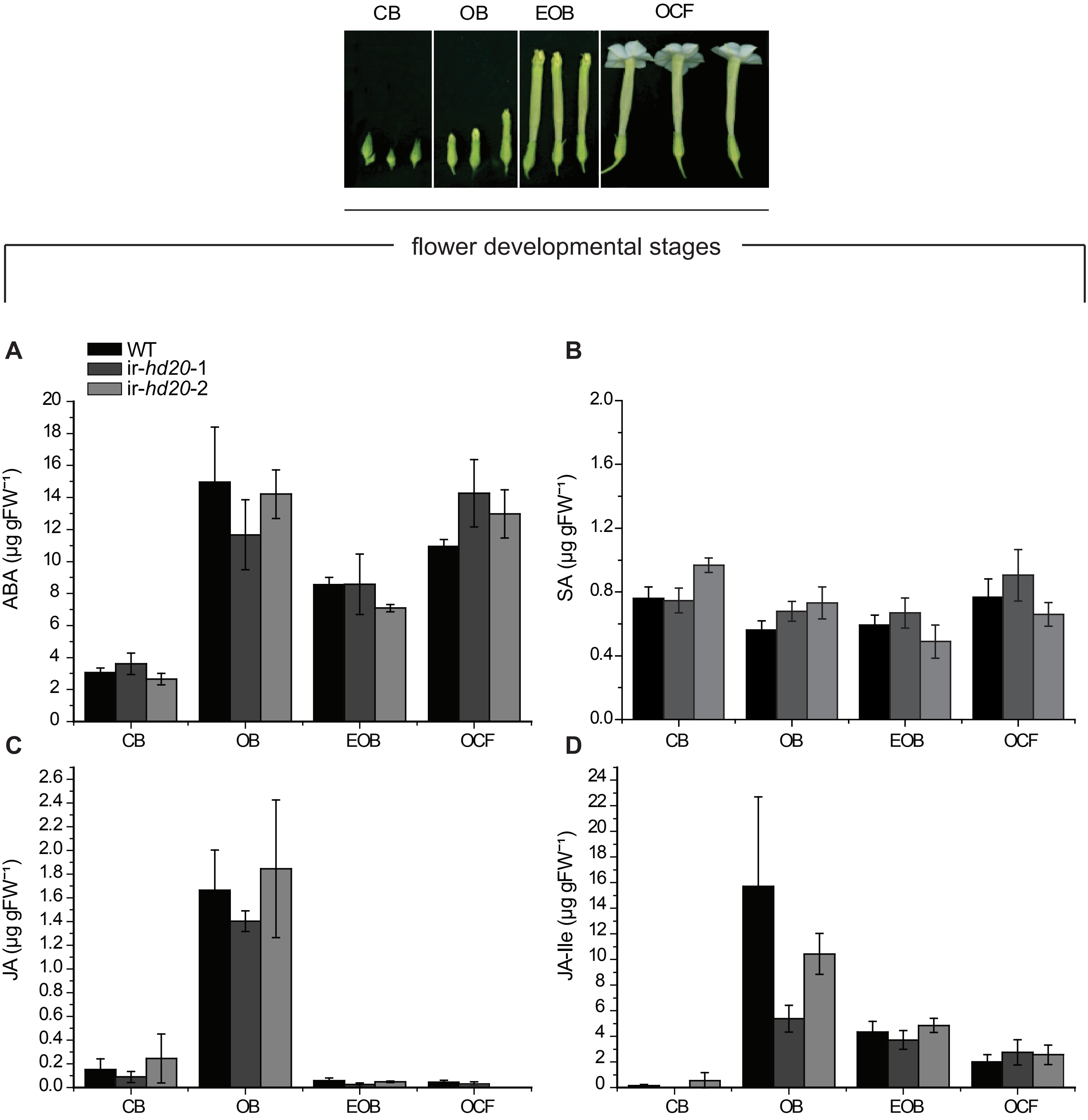

Figure S4. Analysis of phytohormones in whole flowers of ir-*hd20* and WT plants at different developmental stages.

Phytohormones were extracted from whole flowers at different developmental stages and quantified by LC-MS/MS as described in the Materials and Methods section (*n*=4; bars denote ± SE). **(A)** ABA levels. **(B)** SA levels. **(C)** JA levels. **(D)** JA-Ile levels. Closed buds (CB), opened buds (OB), elongating opened buds (EOB), opened corolla flowers (OCF).
