# Supplementary material for: RNAi-mediated silencing of the HD-Zip gene HD20 in Nicotiana attenuata affects benzyl acetone emission from corollas via ABA levels and the expression of metabolic genes
Source: BMC Plant Biol. 2012 May 1;12:60. doi: 10.1186/1471-2229-12-60 (PMC3413612; doi:10.1186/1471-2229-12-60)
Supplement: Additional file 6 — List of primers for qPCR analysis. [file 1471-2229-12-60-S6.pdf]

Additional file 6
List of primers for qPCR analysis

Primer name	Sequence 5'>3'
Na-HD20 Fw	CCGAGAAAGAAGGTGGACAGTATTG
Na-HD20 Rv	AGCCGAATAATCAGCCTTTATGC
Na-Chal1 Fw	TCATTTGGATAGTATGGTCGGG
Na-Chal1 Rv	ACCGTTGATAGCGCCATCGC
Na-CK Fw	TTCTCTGTTACCTGCTTCAACC
Na-CK Rv	CTACACAGAGATCCGAAGGAG
Na-SAMT/BAMT Fw	AAGCAATGGCCAAAAGCAGTCGC
Na-SAMT/BAMT Rv	TTCGTAGAACGCCGGCAACGAC
Na-DFR Fw	GGCAGCGCCAACGGTTTGC
Na-DFR Rv	TCAAGTCCGCTTTCCACAGCGTC
Na-PP2C Fw	TCCCGTCGGCCACTTACCTCC
Na-PP2C Rv	CATGGTTGCTCGCCATCCTTGC
